# Effort-aware and just-in-time defect prediction with neural network

**DOI:** 10.1371/journal.pone.0211359

**Published:** 2019-02-01

**Authors:** Lei Qiao, Yan Wang

**Affiliations:** School of Computer Science and Technology, Beijing Institute of Technology, Beijing, China; Newcastle University, UNITED KINGDOM

## Abstract

Effort-aware just-in-time (JIT) defect prediction is to rank source code changes based on the likelihood of detects as well as the effort to inspect such changes. Accurate defect prediction algorithms help to find more defects with limited effort. To improve the accuracy of defect prediction, in this paper, we propose a deep learning based approach for effort-aware just-in-time defect prediction. The key idea of the proposed approach is that neural network and deep learning could be exploited to select useful features for defect prediction because they have been proved excellent at selecting useful features for classification and regression. First, we preprocess ten numerical metrics of code changes, and then feed them to a neural network whose output indicates how likely the code change under test contains bugs. Second, we compute the benefit cost ratio for each code change by dividing the likelihood by its size. Finally, we rank code changes according to their benefit cost ratio. Evaluation results on a well-known data set suggest that the proposed approach outperforms the state-of-the-art approaches on each of the subject projects. It improves the average recall and popt by 15.6% and 8.1%, respectively.

## Introduction

Software quality assurance activities, such as the defect prediction and source code inspection, have a great influence on producing high quality reliable software [1, 2]. However, such activities are expensive with the increasing scale of modern software since these software systems are complex and failure-prone. In most cases, the developers have limited resources and insufficient time to test the software [3]. To reduce defects and improve the reliability of such software applications, developers should inspect source code changes thoroughly to identify and fix all defects. However, source code inspection is challenging, labor-intensive, tedious and time consuming. In addition, testing all units for finding bugs is impractical because the software program budgets are finite and the release schedules are tight [4].

To facilitate source code inspection, software defect prediction techniques have been proposed [5]. Defect prediction techniques help software quality assurance team to inspect the code area that detect the most likely defective code [6]. Developers utilize the software defect prediction techniques that automatically detect the potential defects in most likely buggy code area to find the bugs and then allocate the limited resources [7]. Consequently, defect prediction plays a critical role in software quality assurance activities. Software defect prediction is to predict how likely a given piece of source code may contain defects (or how many defects it may contain) [8–10]. With such defect prediction techniques, developers rank software modules for inspection. Based on such ranks, developers may invest less effort in inspecting those source code changes that are more likely to contain defects. As a result, more defects can be identified with limited inspection effort.

The process of software defect prediction [4, 11, 12] involves three steps. The first step is to collect history data sets for training [13]. To this end, researchers identify and recover defect-inducing changes by building the link between the source code from Concurrent Versions System (CVS) and bug reports from bug tracking system (e.g., bugzilla) to generate such data sets. The second step is to build a classifier which can predict whether a change is buggy or not [11]. The third step is to make predictions based on the resulting classifier [11]. Defect prediction can be applied at different granularity: packages [14], files [15], methods [16, 17] or changes [13, 18]. Change-level defect prediction is proposed by Mockus and Weiss [19] to recommend which changes made to software applications should be inspected first. It is also known as just-in-time (JIT) defect prediction because the prediction are made promptly once source code changes are committed [20]. JIT defect prediction has many advantages. First, JIT defect prediction can narrow down inspecting source code when find defects. Developers can only review the most likely buggy change without inspecting the whole file or package. The amount of code for inspecting in a change is less than in a file or package. Second, JIT defect prediction can help developers identify whether a change is defect-inducing change at check-in time [21], when the code are still fresh in their minds. JIT defect prediction can find the bugs in time and help the developers allocate the limited test resources reasonably in practical applications.

Most defect prediction approaches take defect prediction as a binary classification problem that could be solved by different classification algorithms [22–26], e.g., Support Vector Machine (SVM) [23], Random Forest (RF) [27] and Nearest-Neighbor [28]. Such approaches simply classify source code changes into two categories: buggy or clean. However, such approaches do not take into account the inspection effort required to discover the predicted defects, i.e., the effort of code inspection on suspicious code changes. As a result such approaches maximize classification performance, e.g., precision, recall, and F-measure, but fail to maximize the number of defects identified with given inspection effort (resource) [29]. To this end, new approaches begin to take into account the inspection effort in defect prediction. Such approaches are also called effort-aware defect prediction where effort is usually measured by the number of modified source code within a given code change [30].

To improve software reliability [31] with limited human resource, in this paper we propose an effort-aware just-in-time defect prediction approach based on neural networks and deep learning. Deep learning has been applied to many learning tasks, such as speech recognition, image processing and code completion [32, 33]. We employ neural network and deep learning in defect prediction because they are good at selecting useful input features automatically, and building the complex mapping between input and output [34, 35]. It is suitable for defect prediction since the mapping between the input (metrics of code changes) and the output (number of defects) is very complex, and we do not know exactly which input feature (metrics) are really useful or useless for defect prediction.

We evaluate the proposed approach on a well-known data set where the number of defects in each code change is identified [13]. The results of the ten-fold cross-validation evaluation suggest that the proposed approach outperforms existing approaches. Compared to the well-known CBS (i.e., Classify-Before-Sorting) [36] and LT (i.e., the unsupervised approach of the lines of code in a file before the change) [18], the proposed approach improves the recall [13] and popt [13, 30] by 15.6% and 8.1%, respectively.

This paper makes the following contributions:

- We propose an effort-aware just-in-time defect prediction approach based on neural network.
- We evaluate the proposed approach with a well-known data set and evaluation results suggest that the approach outperforms existing approaches.

The rest of this paper is summarized as follows. Section 2 introduces the background and related work on defect prediction. Section 3 gives the details about our approach, neural network based regression model (denoted as NNR model for short), including: data preprocessing and the methodology of building NNR model. Section 4 provides the evaluation of our research and the results comparison of our approach with previous studies. Section 5 examines the threats to validity of our research. The conclusion is given in Section 6.

## Related work

### Defect prediction

Software applications are often complex and thus fault-prone. Defect prediction helps developers to locate and fix more bugs quickly with less effort. Akiyama built the first defect prediction model to predict the number of defects based on lines of code (LOC) in 1971 [37]. After that, a number of defect prediction approaches have been proposed [7, 13, 18, 22, 23]. Such approaches can be divided into two categories: supervised approaches and unsupervised approaches. Supervised approaches learn from the history labeled data by training classifiers on such data. To facilitate the research of supervised approaches, Kamei et al. build a data set from six open-source projects by mining source code repositories and bug reports [13]. They propose an effort-aware just-in-time change-level supervised defect prediction approach. They also leverage logistic regression to build a customized supervised logistic regression (LR) model and use the linear regression to build an effort-aware linear regression (EALR) model for the defect prediction. They find that EALR model is better than LR model. Unsupervised defect prediction predicts defect proness without requiring access to training data. Unsupervised defect prediction approaches, such as clustering techniques, are more appropriate for building the models when there is lacking of software defect data [38]. One main advantage of unsupervised model is that it does not need to collect and label historical defect training data. However, the research of unsupervised defect prediction approaches is very scarce. One reason is that unsupervised defect prediction is usually underperforms supervised ones in terms of prediction performance [39]. Yang et al. [18] present an unsupervised approach that ranks every change in descending order according to the reciprocal of the raw value of every change metric. Surprisingly, they find that the unsupervised model based on LT outperforms the state-of-the-art supervised approach EALR. Fu and Menzies [40] replicate and extend Yang et al.’s [18] study, their experiment results show that not all unsupervised models work better than supervised models for effort-aware just-in-time defect prediction.

Defect prediction can be performed on different granularity. Some defect prediction are made at package level [41]. Package-level defect prediction is based on the hypothesis that the likelihood of a package to fail is significantly determined by the problem domain [14]. The package-level defect prediction is to predict failure-prone components on the package-level (e.g., Martin’s metrics) defect history data sets. For example, Mishra et al.[41] propose a new Support Vector based Fuzzy Classification System (SVFCS) for defective module prediction at package level. Some defect prediction are made at method level [16]. Method-level defect prediction is to predict bug-prone methods on the method-level (the McCabe’s and Halstead’s ones) data sets. For example, Giger et al.[16] investigate bug prediction models at the level of individual methods. They decrease the granularity of defect prediction and thus reduce manual inspection efforts. In addition, the change level defect prediction has been proposed as well [13, 18]. It makes prediction using change metrics at check-in time [21] when the code impression is still fresh in developers’ minds. It is performed whenever software changes are submitted to the version control systems. Mouckus and Weiss [19] first propose just-in-time (JIT) defect prediction and they use all the properties of the software changes to build a logistic model for defect prediction. Kamei et.al’s EALR model [13] and Yang et al.’s LT model [18] perform just-in-time defect prediction on change-level as well. Yang et al. apply the deep learning to the JIT defect prediction [42]. They exploit the Deep Belief Network (DBN) that consists of three Restricted Boltzmann’s Machine (RBM) and a logistic regression classifier to build the deeper model. Then, they use the model to detect more expressive features instead of the initial features (i.e., fourteen metrics). Finally, a classifier is built based on the selected expressive features. Given a new change, the classifier can predict the change as buggy or clean based on the confidence scores. In our paper, we use three full connection Back Propagation (BP) neural networks [43] to build a regression model, and not a classifier model. Our neural network regression (NNR) approach exploits 10 change metrics except the number of modified directories (ND), recent developer experience (REXP), the lines of code added (LA) and the lines of code deleted (LD). The output of our neural networks regression approach is a prediction value (i.e., defect probability). Our changes ranking approach is different from Yang et al. We sort our data set according to the predicted defect probability divided by the sum of LA and LD in descending order. Recently, Huang et al. [36] propose the CBS (classify before sorting) approach and conduct defect prediction on change level as well. The core idea of CBS approach is that among the changes classified to be defective, the smaller ones should be inspected first. First, they build a linear classifier following Kamei by using the linear regression. Second, changes are classified into two groups: defective and non-defective by the classifier. Third, They sort the predicted defective changes in ascending order according to their size (i.e., la + ld) to build the CBS model. Current defect prediction techniques mainly depend on historical data. However, historical data from past projects may not be available. Consequently, the defect prediction would be difficult. To address this problem, Li et al. propose sample-based methods for building cost effective defect prediction model while keeping the sample size small [44]. They randomly sample a small percentage of modules from a local project to construct a classification model. Then they use the model to predict defect-proneness of the un-sampled modules in the current project, independent from the past project. Their methods involve three methods, sampling with conventional machine learners, sampling with semi-supervised learning (i.e., the CoForest method) and sampling with active semi-supervised learning (i.e., the ACoForest method). Their approach does not require historical data and the defect prediction is made at package-level and file-level. In contrast, our proposed defect prediction approach requires historical data and is made at change-level.

### Effort-aware defect prediction and performance indicators

The previous studies on defect prediction do not consider the effort of source code inspection [9, 15, 45]. Most studies of defect prediction simply take the defect prediction as a classification problem, and evaluate the performance with precision, recall and F-measure [23]. In other words, traditional defect prediction approaches assume that the effort to test and inspect software modules is constant regardless of the different sizes of the modules [45]. To take the inspection effort into consideration, Mende and Koschke [30] propose an effort-aware defect prediction model that ranks source code based on not only software defect-proneness (i.e., the number of defects or defect probability) but also the inspection effort (often measured by the number of source code to be inspected).

**Fig 1:**
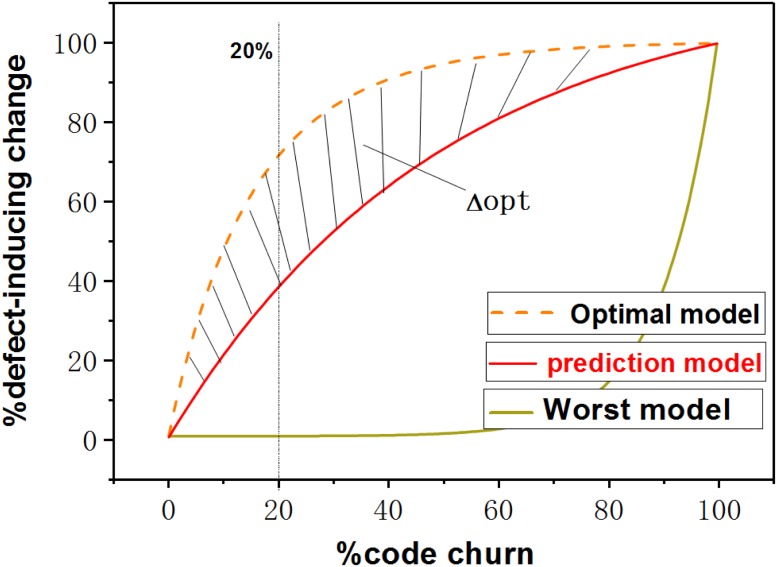
Schematic diagram of an effort-based cumulative lift chart.

To evaluate such effort-aware approaches, Mende et al. [46] propose a new metric Δ*opt*. Δ*opt* is defined as the area between the predicted model and optimal model, which results in a scalar value as shown in Fig 1. In Fig 1, the horizontal axis is the cumulative percentage of code churn of the changes (i.e., the inspection effort). The vertical axis is the cumulative percentage of defect-inducing changes found in selected changes. Δ*opt* depicts deviations between predicted model and optimal model. Based on Δ*opt*, they define *Popt* as follows:

$$\begin{array}{c} Popt=1-\Delta opt \end{array}$$ (1)

The larger *Popt* is (i.e., the smaller Δ*opt* is), the closer the prediction model is to the optimal model. That is, a lager *Popt* means a better performance of the effort-aware JIT defect prediction approach. *Popt* is dependent on the number of defects in the data set. Consequently, normalising the *Popt* for the prediction model is necessary for comparison among different data sets. The normalized *Popt* computational formula can be defined as follows:

$$\begin{array}{c} Popt\left(pre\_model\right)=1-\frac{Area(optimal)-Area(pre\_model)}{Area(optimal)-Area(worst)} \end{array}$$ (2)

*Area*(*optimal*), *Area*(*pre*_*model*), and *Area*(*worst*) represent the area under the curve corresponding to the optimal model, the prediction model and the worst model, respectively.

*Recall* is another performance indicator widely used to measure the performance of the effort-aware defect prediction. It measures how many percentage of defects could be identified when a certain percentage (e.g., 20%) of the effort is allocated. It is calculated as follows:

$$\begin{array}{c} Recall=\frac{m}{n} \end{array}$$ (3)

where *m* represents the number of identified defects, and *n* represents the total number of defects in test data set. *Recall* depends on the allocated inspection effort, and the more effort is allocated, the greater recall will be. The recall value when allocated effort is just enough to inspect 20% of the source code is widely used because research results suggest that 20% of the files (costing 20% of inspection effort) contain around 80% of the defects [47].

### Deep learning in software engineering

There are many deep learning algorithms, such as Deep Belief NetWork (DBN)[48], Recurrent Neural Networks (RNN)[49], Convolutional Neural Networks (CNN)[50] and Long-Short Term Memory (LSTM) [51]. Deep learning has been applied in many fields of software engineering. Raychev et al. [32, 33] use the recurrent neural network (RNN) model to predict the probability of (i + 1)-st word for code completion. White et al. [52] leverage deep software language model (RNN) in code suggestion. Mou et al. [53] proposed a tree-based convolutional neural network (TBCNN) for programming language processing, which can detect the structural features of program. Ling et al. [54] propose an attention model for code generation. Lam et al. [55] propose a new approach that leveraged deep neural network (DNN) in combination with rVSM to improve the accuracy of bug localization.

Deep learning has also been applied in defect prediction [11, 42, 56]. Yang et al. employ Deep Belief NetWork to features detection for defect prediction [42]. They use DBN for advanced features integration from fourteen change metrics. Then, they construct a logistic regression classifier to predict the changes as buggy or clean. Our approach is different from their approaches in that they take the defect prediction as a binary classification problem, whereas we take it as a regression problem. Besides that, they connect the Deep Belief NetWork to a logistic regression classifier whereas our approach is a pare network.

Wang et al. [11] leverage the deep learning technology to learn semantic features directly from source code for defect prediction. They utilize Deep Belief Network to generate semantic features from token vectors extracted from programs’ java Abstract Syntax Tree (AST). After that, they build a defect prediction model and perform defect prediction. They use the semantic features instead of the traditional features (Halstead features [57] and Mood features [58]) for the defect prediction. Our defect prediction use the change metrics that is often selected by experts for defect prediction as well.

Li et al. use Convolutional Neural Networks (CNN) for effective feature generation from source code with semantic and structural information to build a defect prediction model [4]. They adopt word embedding and combine the CNN-learned features with traditional features. However, they regard the defect prediction as a classification problem as well and inspection effort is not considered.

As a conclusion, our approach is different from the existing neural network based defect prediction approaches in the following aspects. First, Wang et al. [11] and Li et al.’s [4] defect prediction approaches are not effort-aware (i.e., their approaches don’t take the effort of code inspection into account) whereas our approach is effort aware. Wang et al. [11] and Li et al.’s [4] approaches take the defect prediction as a binary classification. Our defect prediction approach regard the defect prediction as the ranking problem. The output of our NNR approach is a regression value, i.e., the defect probability of a change. Then we rank the changes according to the defect probability of a change divided by the code churn (i.e., la + ld). Second, Wang et al. [11] and Li et al.’s [4] approaches perform defect prediction on the file-level whereas our defect prediction approach is on the fine granularity (i.e., change-level).

## Approach

### Overview

**Fig 2:**
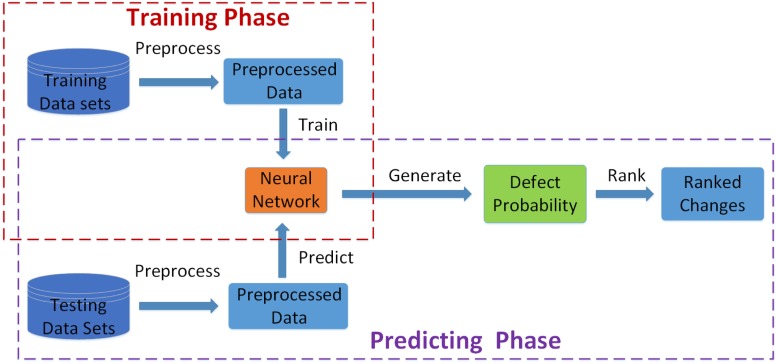
Overview of our proposed approach.

An overview of the proposed approach is presented in Fig 2. The approach is composed of three steps: train a *NNR model*, make defect prediction for new changes based on the resulting *NNR model* and rank code changes for manual code inspection. To train the *NNR model*, we preprocess training data by applying logarithm, normalization and sampling. At prediction phase, we preprocess the metrics of the given code change and feed them into the trained neural network. The network generates the probability for the code change that contains defects. Based on the probability as well as the inspection effort for the code changes, we rank such changes so that inspecting them in order could find more buggy code changes with limited inspection effort. In the following sections, we present each of the key steps of the proposed approach.

### Metrics for code changes

The public data set for JIT defect prediction is available at http://research.cs.queensu.ca/∼kamei/jittse/jit.zip, shared by Kamei [13]. They generate the data set as follows. First, they extract information from the version control system (e.g., CVS repositories) of the projects and combine it with bug reports. Second, they use the heuristics to group related one-files commits into one software change. Third, they use the SZZ algorithm [59] and Approximate SZZ(ASZZ) algorithm to identify and recover the defect-inducing changes. In addition, they process the data as follows. (1) They deal with collinearity by manually removing the most highly correlated factors and using the stepwise variable selection. (2) They deal with skew by applying a standard log transformation. (3) They perform resampling of the training data to deal with data imbalance. They randomly delete the majority class (i.e., non-defect-inducing changes) to make the number of the majority class equal to the number of the minority class (i.e., defect-inducing changes).

The public data set contains 14 metrics of code changes. Such metrics are briefly introduced in Table 1 and they are concerned with five dimensions [13]. The first dimension is diffusion, concerning the number of modified subsystems (NS), the number of modified directories (ND), the number of modified files (NF) and the distribution of modified code across each file (Entropy). The second dimension is size, concerning the lines of code added (LA), the lines of code deleted (LD) and the lines of code in a file before the change (LT). The third dimension is purpose, only concerning FIX. Yin et al.’s [60] research shows that the change to fix a defect is more likely to introduce a new defect and incorrect bug-fixes will also introduce a new defect. FIX is whether or not the change is a defect fix. The fourth dimension is history, concerning the number of developers that changed the modified files (NDEV), the average time interval between the last and the current change (AGE) and the number of unique changes to the modified files (NUC). The fifth dimension is experience, concerning developer experience (EXP), recent developer experience (REXP), and subsystem experience (SEXP). Mockus and Weiss [19] argue that a programmer with higher experience will reduce their likelihood of inducing defect in a change. Developer experience (EXP) is measured by the number of changes made by the developer before current change. Recent developer experience (REXP) is measured by the total experience of the developers in term of change, weighted by their age. Subsystem experience (SEXP) measures the number of changes the developer made in the past to the subsystems that are modified by the current change.

**Table 1:** Metrics of code changes.

Dimension	Metric Name	Description
Diffusion	NS	The number of modified subsystems
	ND	The number of modified directories
	NF	The number of modified files
	Entropy	Distribution of modified code across each file
Size	LA	Lines of code added
	LD	Lines of code deleted
	LT	Lines of code in a file before the change
Purpose	FIX	Whether or not the change is a defect fix
History	NDEV	The number of developers that changed the modified files
	AGE	The average time interval between the last and the current change
	NUC	The number of unique changes to the modified files
Experience	EXP	The developer experience in terms of number of changes
	REXP	Recent developer experience
	SEXP	Developer experience on a subsystem

### Data preprocessing

We preprocess training data by applying logarithm, normalization and sampling.

#### Natural logarithm

Log transformation is widely used to make highly skewed distributions data sets less skewed [61]. It can make data conform to normality, reduce variability of data, and make patterns more visible [61]. We take the natural logarithm of each change metrics with the following formula:

$$\begin{array}{c} x^{{}^{\prime }}=\ln x \end{array}$$ (4)

Note that the transformation is not applied to FIX because it is boolean, i.e., it is either zero (no) or one (yes).

#### Normalization

Data normalization is widely used to transform data values to limit their changing range to a small range, e.g., from zero to one. Normalization is particularly useful for neural networks algorithms because it can reduce the estimation errors and decrease calculation time needed in training process [62]. Normalization can also reduce the chances of getting stuck in locally optima solution. One of the widely used normalization is Min-max normalization [63] that performs a linear transformation on the original data. Min-max normalization is defined as follows:

$$\begin{array}{c} Norm\left(x\right)=\frac{x_{i}-min\left(x\right)}{max(x)-min(x)} \end{array}$$ (5)

where *min*(*x*) and *max*(*x*) represent minimum value and maximum value of a change metric *x*, respectively. *x*_*i*_ represents a raw value of this metric. Min-max normalization maps a value x_*i*_ to *norm*(*x*) in the range [0, 1]. Such normalization can preserve the relationship among the raw data values [64]. It does not change the distribution of the data set.

In our data set, the range of change metrics is not uniform. NS, NM, NF, NDEV, PD, EXP, REXP and SEXP are raw data with different ranges [13]whereas other metrics have been normalized to [0, 1]. To unify the data format, we normalize such raw data with Min-max Normalization. We choose the Min-max normalization data set because of its high accuracy, low complexity and high learning speed [65].

#### Sampling

The training data is imbalanced because most of the changes are defect free whereas only a small percent of changes contain defects. The imbalance might reduce the accuracy of the prediction. To this end, we should ensure that the number of buggy changes is equal to the number of buggy free changes in the training data. We employ under-sampling technique [66] to ensure the equality of the numbers. Under-sampling method uses a subset of the majority class by only removing borderline and noisy majority examples to train the classifier. Since many majority class are ignored, the training set becomes more balanced [66].

### Modelling

The key of the proposed approach is to predict how likely a given code commit contains defects. The prediction could be represented as a mapping:

$$\begin{array}{c} y=f(cm) \end{array}$$ (6)

$$\begin{array}{c} y\in [0,1] \end{array}$$ (7)

where *cm* is the given code commit and *y* is the possibility for *cm* to contain defects.

As introduced in Metrics for Code Changes Section, code commits are often represented as a set of metrics in defect prediction.

$$\begin{array}{cc} x(cm)=<NS(cm),NF(cm),Entropy(cm),LT(cm),FIX(cm), & \left(8\right)\\ NDEV(cm),AGE(cm),NUC(cm),EXP(cm),SEXP(cm)> & \left(9\right) \end{array}$$

where *NS*(*cm*), *NF*(*cm*), *Entropy*(*cm*), *LT*(*cm*), *FIX*(*cm*), *NDEV*(*cm*), *AGE*(*cm*), *NUC*(*cm*), *EXP*(*cm*), *SEXP*(*cm*) are code metrics introduced in Metrics for Code Changes Section. As a result, the model of defect prediction can be represented as:

$$\begin{array}{c} y=g(x(cm)) \end{array}$$ (10)

Up to date, the mapping *g* is not yet full understood. Existing approaches [13] employs statistics based machine learning techniques (e.g., linear regression) to search for such mappings that best fit a given corpus of labelled data. In this paper, we try to learn the mapping with deep learning techniques as introduced in the following section.

### Neural networks based defect prediction

**Fig 3:**
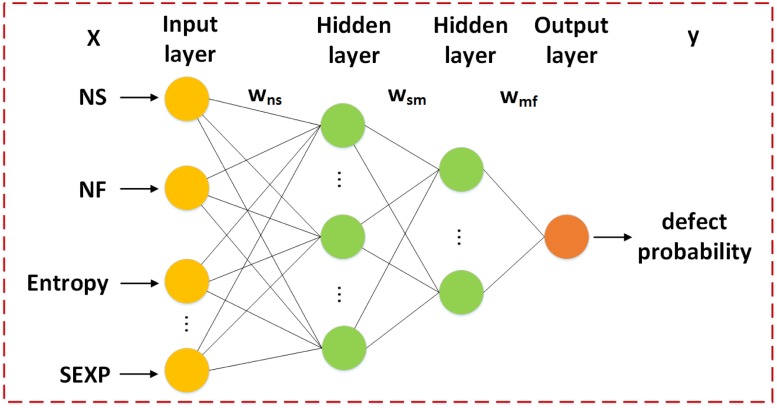
Overview of the neural network.

We propose a neural network and deep learning based approach for effort-aware JIT defect prediction. The neural network is presented in Fig 3. It’s a fully connected neural network. It consists of one input layer, one hidden layer and one output layer. *X* = 〈*NS*, *NF*, …*SEXP*〉 is the input of the neural network, *y* is the predicted value of fully connected neural network (i.e., defect probability), and *w*_*ns*_, *w*_*sm*_…*w*_*mf*_ are the weights for different input of the neurons. In total we have a connected neural network with three *dense* layers. We use ten change metrics (i.e., all fourteen change metrics except ND, REXP, LA and LD) as the input of the neural network. It’s output is the defect probability for changes. Consequently, the dimension of the input of the neural network is 10 and the dimension of output is 1. For the first layer, we set input_dim = 10, output_dim = 20, kernel_initializer = ‘uniform’ and activation function = ‘tanh’. For the second layer, we set output_dim = 10, kernel_initializer = ‘normal’ and activation function = ‘relu’. For the last layer, out_dim = 1 and kernel_initializer = ‘normal’. We preprocess ten change metrics (i.e., all fourteen change metrics except ND, REXP, LA and LD) and feed them to the input layer of the neural network. NF and ND, REXP and EXP are highly correlated, high correlated features may decrease the performance of defect prediction. So we exclude the ND and REXP and use the change metrics NF and EXP instead. LA and LD are highly correlated. Nagappan and Ball [67] argue that relative churn metrics perform better than absolute metrics when predicting defect density. So, we remove the change metrics LA and LD and they will be used for ranking. The output of the neural network is the defect probability of the input changes. We employ Back Propagation (BP) algorithm to tune the weights with training data.

We use the training data to train the model. After that, we get a resulting model in each iteration of the k-fold cross validation. The mean_squared_error of the resulting models varies from 0.043 to 0.014. The mean_absolute_error ranges from 0.067 to 0.045 suggesting that the resulting models are quite accurate. To facilitate replication, we publish the source code of our model and the trained model at https://github.com/donaldjoe/Effort-Aware-and-Just-in-Time-Defect-Prediction-with-Neural-Network.

### Effort-aware ranking

We compute the ranking benefit cost ratio *BCR*(*c*) for a given change as follows:

$$\begin{array}{c} BCR\left(c\right)=\frac{P(c)}{Effort(c)} \end{array}$$ (11)

where *P*(*c*) is defect probability predicted by the neural network, *Effort*(*c*) is the required inspection effort measured by code churn (i.e., la + ld)[13]. For a set of changes, we rank them according to their *BCR*(*c*) in descending order.

## Evaluation

To validate the model, we follow Kamei et al.[13], Yang et al.[18] and Huang et al.[36] to conduct a 10-fold cross-validation technique as data analysis method and divide the data set into training data and testing data. In this section, we present the evaluation of the proposed approach on a publically available data set. The major purpose of the evaluation is to investigate whether the proposed approach can outperform existing approaches. To this end, we compare the proposed approach against the state-of-the-art approaches, i.e., EALR model, LT model and CBS model. Kamei et al.’s EALR model, Yang et al.’s LT model, and Huang et al.’s CBS model are the well-known defect prediction approaches. Such approaches are the just in time (i.e., make defect prediction on the change level) and effort-aware (i.e., taking the effort of code inspection into account) defect prediction approaches that are compared to the proposed approach.

### Subject applications

We evaluate the selected approaches on six open-source subject projects, i.e., Bugzilla, Columba, Eclipse JDT, Eclipse Platform, Mozilla and PostgreSQL. We perform our effort-aware JIT defect prediction approach on a well-known data set created by Kamei et al. [13] (http://research.cs.queensu.ca/kamei/jittse/jit.zip). The data set is composed of changes from six open-source projects, i.e., Bugzilla (http://msr.uwaterloo.ca/msr2007/challenge/index), Columba (rsync://columba.cvs.sourceforge.net/cvsroot/columba/), JDT (http://msr.uwaterloo.ca/msr2008/challenge/index.html), Platform (http://msr.uwaterloo.ca/msr2008/challenge/index.html), Mozilla (http://msr.uwaterloo.ca/msr2007/challenge/index) and PostgreSQL (rsync://anoncvs.postgresql.org/cvsroot/pgsql/). The data for Bugzilla and Mozilla are provided by MSR 2007 Mining Challenge, the data for Eclipse JDT and Platform are provided by MSR 2008 Mining Challenge, and the data for Columba and PostgreSQL are generated by combining the changes from the CVS repositories and bug reports from the bug tracking system (i.e., bugzilla) [13]. The data set is created by Kamei [13] and used by Yang et al. [18], and Huang et al. [36].

Table 2 gives the statistics of the six projects. The first column is the subject project name. The second column is the period time of the changes. From the third column to the sixth column present the total changes, percentage of defect-inducing defects, average of LOC per change, and number of the files modified in a code change, respectively. The language of the projects involves Perl, Java, C++, and Rubby. The number of total changes ranges from 4455 to 98275. The percentage of defects ranges form is 5% to 36%.

**Table 2:** Statistics of the studied data sets.

Project	Period	Total Changes	% of Defects	Mean LOC per change	Modified Files per Change
Bugzilla	08/1998–12/2006	4620	36%	37.5	2.3
Columba	05/2001–12/2007	4455	31%	149.4	6.2
Eclipse JDT	05/2001–12/2007	35386	14%	71.4	4.3
Eclipse Platform	05/2001–12/2007	64250	14%	72.2	4.3
Mozilla	01/2000–12/2006	98275	5%	106.5	5.3
PostgresSQL	07/1996–05/2010	20431	25%	101.3	4.5

### Process

**Fig 4:**
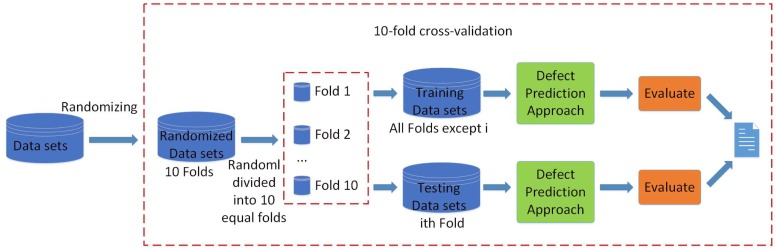
Process of the evaluation.

An overview of our experiment process of evaluation is shown in Fig 4. 10-fold cross-validation technique [68] has been used in previous studies [13, 18, 36]. In consistent with previous studies, in our paper, we adopt 10-fold cross-validation technique to evaluate the performance of our defect prediction approach (i.e., NNR) against the state-of-the-art approaches (i.e., EALR, LT, and CBS) on the data set of each subject project. The process is presented in Fig 4. First, we preprocess the data set (it has been discussed in Data Preprocessing Section, i.e., applying logarithm, normalization and sampling). Second, we randomize the data set and randomly divide the data of each subject project into ten equal subsets. At each fold of the 10-fold cross-validation, we use one subset of the data as testing data (*testingData*) and the remaining nine subsets as training data (*trainingData*). Each subset is used as testing data once. For each fold, the evaluation is conducted as follows:

1. First, we train the proposed approach (i.e., NNR) with *trainingData* and get the *trainedModel*.
2. Second, we evaluate the *trainedModel*, EALR, LT and CBS on *testingData* independently.
3. Third, we calculate recall and popt value for each of the evaluated approaches.

The whole process is repeated 10 times in total and has 10 prediction value to diminish the bias in the data set splits. The average recall and popt value for each approach is calculated.

Recall is calculated according to Eq 3 (in Effort-Aware Defect prediction and Performance Indicators Section). Popt is calculated according to Eqs 1 and 2 (in Effort-Aware Defect prediction and Performance Indicators Section).

We run the experiments on a personal computer: Intel Core i7-6700 CPU 3.4GHz, 16GB RAM. We use keras (http://keras.io) to build our neural network model.

### Results

We carry out 10-fold cross-validation on the data set and compare the proposed approach against the state-of-the-art approaches, i.e., EALR [13], LT [18], and CBS [36].

**Fig 5:**
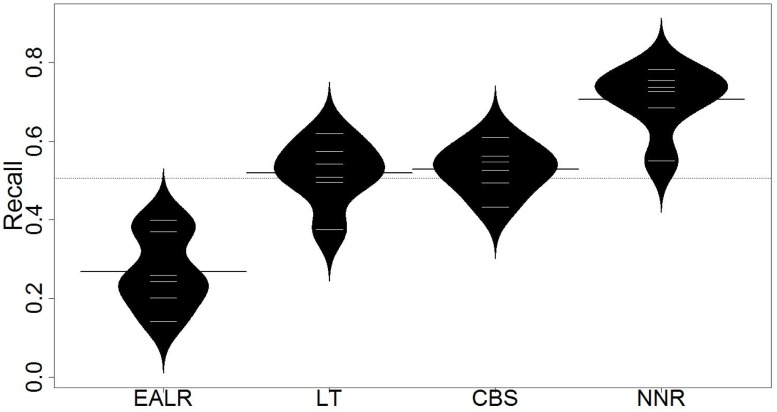
Distribution of recall (beanplot).

**Fig 6:**
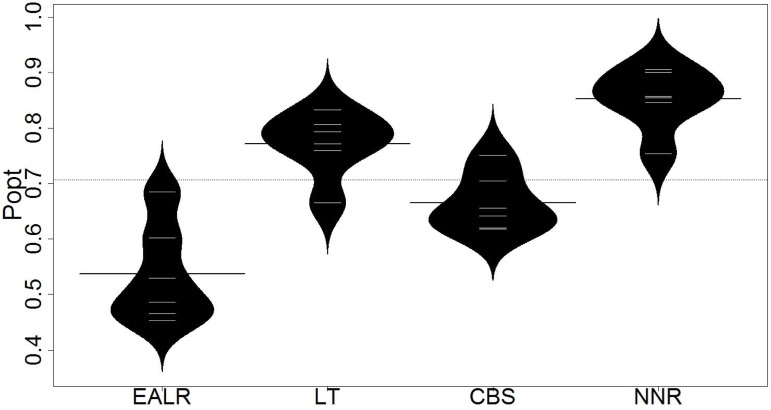
Distribution of popt (beanplot).

Evaluation results are presented in Tables 3 and 4. Each row of the tables represents a subject application except that the last rows represent the average performance on all subject applications. Columns of these tables represent different approaches, i.e., EALR, LT, CBS and NNR (the proposed approach). Table 3 presents the recall of the approaches on six open-source projects. The recall is calculated when the allocated effort is just enough to inspect 20% of the source codes. The highest recall on each project is shown in bold. Table 4 presents the popt value of the approaches on six open-source projects. The highest popt are highlighted in bold as well. The distribution of recall and popt are depicted in Figs 5 and 6 respectively.

**Table 3:** Recall of the approaches.

Project	EALR	LT	CBS	Our Approach
Bugzilla	37.0%	49.5%	56.2%	**75.4%**
Columba	39.9%	62.0%	52.6%	**78.3%**
Eclipse JDT	20.1%	57.4%	54.8%	**72.7%**
Eclipse Platform	25.9%	50.9%	61.0%	**73.7%**
Mozilla	14.1%	37.5%	43.3%	**55.0%**
PostgresSQL	24.3%	54.2%	49.4%	**68.5%**
Average	26.9%	51.9%	53.1%	**69.6%**

**Table 4:** Popt of the approaches.

Project	EALR	LT	CBS	Our Approach
Bugzilla	68.5%	76.0%	75.1%	**90.1%**
Columba	60.2%	83.3%	64.2%	**90.6%**
Eclipse JDT	46.6%	79.4%	65.6%	**85.8%**
Eclipse Platform	53.0%	77.2%	70.5%	**85.5%**
Mozilla	45.3%	66.5%	61.8%	**75.4%**
PostgresSQL	48.6%	80.7%	62.0%	**84.6%**
Average	53.7%	77.2%	66.2%	**85.3%**

From such tables and figures, we make the following observation:

- First, the proposed approach (i.e., *NNR*) outperforms the state-of-the-art approaches (i.e., EALR, LT, CBS) in both recall and *popt*. The proposed approach improves the average recall by 15.6% compared to the state-of-the-art approaches. The average recall achieved by EALR, LT, CBS and NNR is 26.9%, 51.9%, 53.1% and 69.6% respectivley. The proposed approach also improves the average *popt* by 8.1% compared to the state-of-the-art approaches. The average *popt* achieved by EALR, LT, CBS and NNR are 53.7%, 77.2%, 66.2% and 85.3% respectivley.
- Second, the proposed approach outperforms the state-of-the-art approaches on each of the subject projects. Generally, the results suggest that the proposed approach outperforms the others regardless of the involved projects.

## Threats to validity

A threat to the construct validity is the suitability of the performance evaluation metrics (recall and popt) of the defect prediction approach. They are employed because they have been widely used in previous effort-aware JIT defect prediction and haven been proved to be effective [13, 18].

A threat to the internal validity is the way we set the cut-off value. The value indicates the allocated inspection effort. It’s represented as the percentage of source code that could be inspected with the allocated inspection effort. Consequently, the greater the cut-off value is, the greater recall would be. In our paper, we use 0.2 as the cut-off value to calculate the recall. The reasons are explained as follows. First, it is widely used by existing approaches [13, 18]. Second, Ostrand et al. found that around 20% lines of code may contain 80% fault [47]. Consequently, inspecting 20% of the most error-pron source code can hopefully find 80% of the defects.

The first threat to external validity is that the data set we used may be incomplete. This may affect the accuracy of our experimental results. The data set we used in our study was generated by Kamei et al. [13]. Kamei et al. [13] establish links between every defect fix and the source code change by using SZZ algorithms [69] and approximate SZZ (ASZZ) algorithms to generate the data sets. The SZZ and ASZZ algorithms may miss some links. The defect-inducing changes found may be not complete [70]. There may be some errors in the processing of extraction of defect-inducing changes. Despite all these, we still use this data set because the data set is the only public available change-level data set and it is widely used in the effort-aware JIT defect prediction.

The second threat to external validity is the limited number of subject applications. We evaluate our proposed approach on six open-source projects. Our study results may not be generalized to the other software projects.

## Conclusion

In this paper, we propose a deep learning based approach for effort-aware just-in-time defect prediction. Based on sample changes, the approach trains a neural network to predict how likely a given change may contain defects. Given a set of changes to be inspected, the proposed approach ranks such changes according to their risky of defects (predicted by the trained neural network) divided by the required inspection effort (measured by the number of lines modified by the change). The key idea of the proposed approach is that we can exploit neural network and deep learning to select useful features for defect prediction because they have been proved excellent at selecting useful features for classification and regression.

Evaluation results on a well-known data set suggest that the proposed approach outperforms the state-of-the-art approaches. It improves the average recall and popt by 15.6% and 8.1%, respectively. We also notice that it outperforms the state-of-the-art approaches on each of the subject projects.
